# Development of Biomimetic Alginate/Gelatin/Elastin Sponges with Recognition Properties toward Bioactive Peptides for Cardiac Tissue Engineering

**DOI:** 10.3390/biomimetics5040067

**Published:** 2020-12-11

**Authors:** Elisabetta Rosellini, Denise Madeddu, Niccoletta Barbani, Caterina Frati, Gallia Graiani, Angela Falco, Costanza Lagrasta, Federico Quaini, Maria Grazia Cascone

**Affiliations:** 1 Department of Civil and Industrial Engineering, University of Pisa, Largo Lucio Lazzarino, 56126 Pisa, Italy; niccoletta.barbani@unipi.it (N.B.); maria.grazia.cascone@unipi.it (M.G.C.); 2 Department of Medicine and Surgery, University-Hospital of Parma, Via Gramsci 14, 43126 Parma, Italy; denise.madeddu@unipr.it (D.M.); caterina.frati@libero.it (C.F.); gallia.graiani@unipr.it (G.G.); angela.falco@unipr.it (A.F.); lagrasta@unipr.it (C.L.); federico.quaini@unipr.it (F.Q.)

**Keywords:** GRGDSP, YIGSR, molecular imprinting, functionalization, cardiac progenitor cells

## Abstract

In recent years, there has been an increasing interest toward the covalent binding of bioactive peptides from extracellular matrix proteins on scaffolds as a promising functionalization strategy in the development of biomimetic matrices for tissue engineering. A totally new approach for scaffold functionalization with peptides is based on Molecular Imprinting technology. In this work, imprinted particles with recognition properties toward laminin and fibronectin bioactive moieties were synthetized and used for the functionalization of biomimetic sponges, which were based on a blend of alginate, gelatin, and elastin. Functionalized sponges underwent a complete morphological, physicochemical, mechanical, functional, and biological characterization. Micrographs of functionalized sponges showed a highly porous structure and a quite homogeneous distribution of imprinted particles on their surface. Infrared and thermal analyses pointed out the presence of interactions between blend components. Biodegradation and mechanical properties appeared adequate for the aimed application. The results of recognition tests showed that the deposition on sponges did not alter the specific recognition and binding behavior of imprinted particles. In vitro biological characterization with cardiac progenitor cells showed that early cell adherence was promoted. In vivo analysis showed that developed scaffolds improved cardiac progenitor cell adhesion and differentiation toward myocardial phenotypes.

## 1. Introduction

Peptide-modified scaffolds are receiving a great amount of attention as support devices for tissue engineering applications [1]. Scaffolds containing peptide sequences offer to seeded cells a biomimetic environment, mimicking the biomolecular signals of the extracellular matrix (ECM). In native tissues, the interactions between cell surface receptors and bioactive peptide sequences exposed by ECM proteins regulate cell behavior by influencing cell adhesion, proliferation, and differentiation. Many bioactive peptide motifs have been identified over the past years to be used for scaffold functionalization [2]. The inclusion of peptide sequences into biomaterials is traditionally achieved by surface modification or by bulk incorporation [3].

Molecular imprinting technology is a totally new approach, originally proposed by our laboratory [4,5], to obtain peptide-modified matrices for tissue engineering. Molecularly imprinted particles with recognition properties toward a peptide sequence of interest (the so-called template) can be obtained through an appropriate copolymerization process, carried out in the presence of a functional monomer, the peptide chosen as template, and a cross-linker. The removal of the template after the polymerization process results in the formation of imprinted cavities, which are physically and chemically complementary toward the template. According to the “epitope approach”, proposed by Rachkov and colleagues, when the imprinted particles will come in contact with the whole protein (containing the peptide used as template), this last will be recognized and rebound [6]. Therefore, after deposition on the scaffold, when the imprinted particles will be exposed to the biological environment or cell culture environment, they will produce an enrichment of a desired ECM protein on the scaffold. Consequently, cell receptors could interact with the rebound protein, promoting a desired cellular response. The feasibility of such approach, as well as the biocompatibility of the imprinted particles, have been already demonstrated in two previous works [4,5].

Molecular Imprinting can offer several advantages with respect to traditional functionalization strategies, such as the covalent attachment of proteins or peptides. Imprinted particles can be deposited on all types of scaffolds, and the presence of specific functional groups for peptide covalent binding is not required. Additionally, the deposition of imprinted particles on the scaffold can be achieved without the use of chemical reagents or solvents, which may induce cell damage. Moreover, the bioactive molecule used as template (usually expensive) could be recovered during the extraction process and reused. Furthermore, if the ambitious goal of a complete removal of the template is achieved, a functional scaffold free of bioactive molecules can be obtained. This could represent a significant advantage from a regulatory point of view, because such a scaffold would fall in the medical device category.

In this work, new molecularly imprinted particles with recognition properties toward two different peptides from ECM proteins were synthesized and characterized. The peptide sequences chosen as templates are H-Gly-Arg-Gly-Asp-Ser-Pro-OH (GRGDSP) from fibronectin and H-Tyr-Ile-Gly-Ser-Arg-OH (YIGSR) from laminin.

Laminin and fibronectin are the main noncollagenous ECM proteins in connective tissues endowed with the ability to regulate cellular phenotype. In particular, fibronectin, the prototype of cell adhesive proteins, has been shown to modulate cell growth, cell shape, cytoskeletal organization, differentiation, migration, and the apoptosis of almost all cells [7,8,9]. Laminins are a large family of glycoproteins, which are the most bioactive components of basement membranes, playing a crucial role in the survival and differentiation of adherent cells [10,11].

The peptide sequences from fibronectin and laminin, used as templates in this work, have demonstrated ability to promote cell adhesion [12]. In addition, the GRDGSP sequence is able to stimulate integrins, such as α5β1 and αvβ3, that are relevant in early cardiac development [13,14], and the YIGSR sequence can increase the ability of stem cells to differentiate into beating cardiomyocytes [15,16,17].

While GRGDSP-imprinted particles were synthetized for the first time in this study, imprinted particles with recognition properties toward the YIGSR sequence were already reported in a previous work [5]. Here, however, with the aim to improve the rebinding performance, a different cross-linker was used during particle synthesis.

Imprinted particles underwent morphological, physicochemical, and functional characterization to assess their recognition and rebinding capability.

Then, both GRGDSP- and YIGSR-functionalized scaffolds were implanted to the rat heart for cardiac tissue engineering. An innovative combination of three natural polymers (alginate/gelatin/elastin, hereafter indicated as AGE) was used as scaffold material to mimic the composition as well as the interactions between components in the native cardiac extracellular matrix [18]. A complete morphological, physicochemical, mechanical, functional, and biological characterization was performed on functionalized sponges.

## 2. Materials and Methods

### 2.1. Materials

Methacrylic acid (MAA > 99%), from Sigma Aldrich (Milan, Italy), was purified by distillation in vacuum to remove the polymerization inhibitor. Azobis(isobutyronitrile) (AIBN > 98%), pentaerythritol triacrylate (PETRA), trifluoroacetic acid (> 99.9%), and phosphate-buffered saline (PBS) were from Sigma Aldrich (Milan, Italy) and used as supplied. Acetonitrile (ACN > 99.9%) from Carlo Erba Reagenti (Milan, Italy) was of HPLC grade purity. The peptides H-GRGDSP-OH and H-YIGSR-OH were from Cambridge Research Biochemicals (Billingham, Cleveland, United Kingdom) and used as supplied. Alginate (viscosity of 2% solution at 25 °C = 250 cps), gelatin (type B from bovine skin), elastin (from bovine neck ligament), and glutaraldehyde (GTA, 25% aqueous solution) were acquired from Sigma Aldrich (Milan, Italy). Calcium chloride was purchased from Carlo Erba Reagenti (Milan, Italy).

### 2.2. Imprinted Particles: Synthesis Procedure

Two groups of molecularly imprinted particles, with recognition properties toward GRGDSP peptide sequence (hereafter indicated as MIP-GRGDSP) and YIGSR peptide sequence (hereafter indicated as MIP-YIGSR), were prepared using a precipitation polymerization method, at the compositions shown in Table 1, following a procedure already reported in the literature [4,5].

Briefly, the template molecule was dissolved in a 70/30 (v/v) ACN/bi-distilled water (ddH_2_O) solution and introduced into borosilicate glass tubes (40 mL). The functional monomer (MAA), the cross-linker (PETRA), and the initiator (AIBN) were added to the polymerization solution. The tubes were sealed under dry nitrogen. The polymerization was thermally initiated at 60 °C and carried out for 20 h under constant stirring. At the end of the polymerization process, the residual polymerization solution was removed and subjected to chromatographic analysis. Collected particles were washed with ACN/ddH_2_O mixture to remove the residual monomer, cross-linker, and not-imprinted template. The template molecule was subsequently extracted by repeated washing with PBS, under vigorous stirring, in order to create the recognition sites.

Control particles (CP) were prepared with the same procedure of the imprinted ones but in the absence of the template molecule.

### 2.3. Alginate/Gelatin/Elastin Sponges: Preparation and Functionalization

AGE sponges were prepared following a procedure similar to that previously used by us to fabricate alginate/gelatin sponges [18]. Briefly, alginate, gelatin and elastin 2% (w/v) bi-distilled water solutions were prepared separately at 50 °C.

Different volumes of natural polymer solutions were mixed together, under stirring at room temperature, in order to obtain an AGE blend with a A:G:E = 10:80:10 weight ratio.

A known volume of the blend was poured into polystyrene Petri dishes and freeze-dried. Then, the obtained sponges were treated with GTA vapors at 37 °C for 18 h for proteins cross-linking and then immersed for 30 min in a solution containing 2% (w/v) CaCl_2_ in bi-distilled water for polysaccharide cross-linking.

After cross-linking treatments, samples were immersed for 16 h in a coagulation bath (0.5 M acetic acid) to promote ionic interactions among the components.

Since GTA and acetic acid could be dangerous for cells, cross-linked sponges were washed repeatedly with bi-distilled water to remove excess GTA and acetic acid, until UV-spectrophotometric and pH analysis of wash waters did not reveal any residual trace.

At the end of the washing procedure, the scaffolds were freeze-dried again.

Sponges were functionalized by the deposition of MIP on their surface. According to the literature, the minimal ligand spacing for cell adhesion in two-dimensional cultures turns out to be 440 nm [19]. This value was extrapolated to three dimensions (calculations based on a body centered cubic unit cell) and correlates to a ligand density of approximately 34 × 10^−12^ mol ligand/cm^3^ [20]. It was decided to modify the sponges using an amount of MIP offering a ligand density at four orders of magnitude above this reference value. In this calculation, both peptide cavities and non-extracted peptides were considered. The calculated quantity of MIP was dispersed in bi-distilled water and deposited on preformed scaffolds.

### 2.4. Morphological Analysis by Scanning Electron Microscopy

The morphology of imprinted and control particles and of AGE sponges, before and after functionalization, was examined by a JSM 5600 scanning electron microscope (SEM, Jeol Ltd., Tokyo, Japan). Before analysis, samples were coated with a gold layer (thickness of 200–500 Å) and mounted on metal stubs. The percentage of porosity and the average pore size were measured analyzing SEM images by the Image J software (National Institutes of Health). The percentage of porosity was calculated from the ratio between the pore area and the total scaffold area.

### 2.5. Chromatographic Analysis

The percentage of monomer conversion during MIP synthesis was determined by high-performance liquid chromatography (HPLC, 200 Series HPLC system, Perkin Elmer, Waltham, Massachusetts, United States), with a UV/VIS detector, following a procedure similar to that already described in previous papers [4,5]. In order to determine the monomer residual amount in the polymerization solution, an Alltima C18 5u column (250 mm length × 4.5 mm i.d.) was used. The mobile phase was A = 0.085% (w/v) trifluoroacetic acid in ACN; B = 0.1% (w/v) trifluoroacetic acid in water. The elution condition was an isocratic elution for 15 min with a mobile phase composition of 54% A and 46% B at a flow rate of 1 mL/min. The injection volume was 100 µl. The detector wavelength was set at λ = 215 nm. 

The amount of template entrapped by the polymeric particles and the amount of extracted template were determined by HPLC measuring the corresponding residual amount in the polymerization solution and in the extraction solution, respectively. To determine the peptide concentrations, a Prosphere HP C4 300 Å 5u column (250 mm length × 4.5 mm i.d.) was used. The elution condition was a linear binary gradient at a flow rate of 1 mL/min, and the gradient was from 30% A and 70% B to 60% A and 40% B in 15 min. The injection volume was 100 µl. The detector wavelength was set at λ = 280 nm.

### 2.6. Infrared Spectroscopy

Fourier transformed infrared spectroscopy (FT-IR) spectra were recorded on a Spectrum-One instrument, Perkin Elmer (Waltham, Massachusetts, United States). The spectrometer was equipped with an attenuated total reflectance (ATR) objective lens with a penetration depth of less than 1 µm. All spectra were obtained at 4 cm^−1^ and represented the average of 16 scans. Spectral images were also acquired using the infrared imaging system of the instrument (Spotlight 300, Perkin Elmer, Waltham, Massachusetts, United States). The spectral resolution was 4 cm^−1^. The spatial resolution was 100 × 100 µm.

### 2.7. Recognition and Selectivity Tests

Recognition and selectivity tests were carried out for MIP-GRGDSP, MIP-YIGSR, and relative controls, CP. The procedure followed was the same, but the composition of the rebinding solution was varied, as described hereafter.

To evaluate the rebinding capacity of MIP, 10 mg of MIP and 10 mg of CP samples were put in contact with 1 mL of a rebinding solution consisting of a template solution (0.25 mg/mL) in water/ACN 30/70 (v/v). The template of the rebinding solution was GRGDSP for MIP-GRGDSP and relative control and YIGSR for MIP-YIGSR and relative control. The tubes, containing the rebinding solution and MIP or CP, were maintained under constant stirring for 30 min. The supernatant was separated from the polymer by centrifugation for 30 min at 14,000 rpm. The procedure was repeated three times, replacing every time the supernatant with fresh solution. Both for MIP and for CP, the amount of rebound template was determined on three samples, and the average values were reported. The residual peptide concentration in the rebinding solution was determined by HPLC at a wavelength of 280 nm.

The amount of template rebound by MIP was calculated as follows:

$$Quantitative\;binding\;\left(\frac{\mu mol\;template}{g\;of\;polymer}\right)=\frac{V\;\times \left(C_{i}-C_{f}\right)}{g\;of\;polymer}$$

where *V*, *C_i_*, and *C_f_* represent, respectively: the volume of the rebinding solution, the initial rebinding solution concentration, and the rebinding solution concentration after adsorption.

The imprinting effect was evaluated through the rebinding factor, which was calculated according to the following equation:

$$\alpha_{MIP/CP}=\frac{\mu mol\;of\;template\;rebound\;by\;MIP}{\mu mol\;of\;template\;rebound\;by\;CP}.$$

When the value of the rebinding factor is higher than one, the recognition capability of MIP is demonstrated.

Selectivity was also verified by incubating fixed amounts of both MIP and CP with 0.25 mg of a template analogue in 1 mL of water/ACN 30/70 (v/v) and then proceeding as for rebinding experiments. The molecules used as analogue were YIGSR for MIP-GRGDSP and GRGDSP for MIP-YIGSR. The selectivity factor *α*’ was calculated as indicated in the equation below:

$$\alpha^{\prime }{}_{\frac{template}{analogue}}=\frac{template\;bound\;by\;MIP}{analogue\;bound\;by\;MIP}.$$

Particles selectivity is demonstrated when *α*’ is higher than 1.

Recognition experiments were also performed on MIP-modified sponges to evaluate if the rebinding capacity was maintained by MIP after deposition on the scaffolds. The procedure followed was the same used to test MIP recognition: 1 cm^2^ of each MIP-modified sponge was put in contact with 1 mL of the rebinding solution. Scaffolds modified with CP were used as controls.

### 2.8. Thermal Analysis

The thermal properties of AGE sponge were studied by carrying out differential scanning calorimetry (DSC) and thermogravimetric analysis (TGA). The DSC thermogram of the sponge was acquired and compared with those of pure alginate, gelatin, and elastin. 

A known amount of the scaffold (about 10 mg) was encapsulated in standard aluminum pans and used for the study. All tests were carried out in triplicate. The samples were heated/cooled/heated at a heating rate of 10 °C/min between −60 and 300 °C, under inert nitrogen. The endothermic peaks were measured using the DSC software.

TGA allowed us to study the thermal degradation process of the three pure polymers and their blend. Samples of ca. 10 mg, in triplicate, were heated from 30 to 700 °C at a rate of 10 °C/min, under nitrogen flow.

### 2.9. Mechanical Analysis

A dynamic mechanical analyzer (DMA8000, Perkin Elmer, Waltham, Massachusetts, United States) was used to investigate the viscoelastic properties of the scaffold. A protocol similar to that described in our previous papers [18,21] was followed. Briefly, the sponge was cut in the form of rectangular strips (20 mm in length, 10 mm in width, 5 mm in thickness). Tests were performed under wet conditions. Before analysis, samples were maintained for 3 h in a solution simulating body fluids (PBS) at 37 °C. Wet conditions were reproduced during analysis. The samples were tested in a single cantilever mode. Storage modulus (E’), loss modulus (E’’), and tangent delta (tan δ) were evaluated by performing a single strain/multi-frequency test. The applied strain had an amplitude of 10 µm; frequencies were set at 1, 3.5, and 10 Hz, reproducing, respectively, a healthy human heart rate (1 Hz, corresponding to 60 bpm), a pathological human heart rate (3.5 Hz, corresponding to 210 bpm), and a supraphysiological pulse rate (10 Hz, corresponding to 600 bpm) [18].

### 2.10. Degradation Test

In vitro hydrolytic degradation tests were performed in PBS, in agreement with the ISO norm 10993-13 “Biological evaluation of medical devices. Part 13: Identification and quantification of degradation products from polymeric medical devices” [22].

Scaffold weight loss during degradation was measured by changes in dry weight after incubation for specified time periods. All the experiments were done in triplicate; the results are the mean (±SE) of three determinations. A separate container was used for each sample.

Samples were dried at room temperature to a constant mass. The starting dry weight, *W*_0_, was determined for each sample by using a balance with adequate precision. Then, samples were fully immersed in a closed container, together with the degradation solution, and maintained in an agitating bath at 37 ± 1 °C. At appointed times, samples were removed from the degradation solution. The samples were rinsed in ddH_2_O and dried to a constant mass. The dry weight at time *t* of degradation, *W_t_*, was determined for each sample. The percentage of remaining weight was evaluated according to the following equation:

$$Remaining\;weight\;\left(\%\right)=\frac{W_{t}}{W_{0}}\times 100.$$

### 2.11. Swelling Test

The swelling properties of the scaffold were evaluated by exposure to aqueous vapor. Samples cut into squares of 1 cm^2^, were dried in oven at 37 °C up to a constant weight and the starting dry weight, *W_d_*, was registered for each sample. Then, samples were introduced in a closed container, with 100 mL of water in the lower part. The container was maintained in an oven at 37 °C for all the duration of the test. At fixed times, the swollen weight, *W_s_*, was determined for each sample. The percentage of swelling was calculated as:

$$Swelling\;\left(\%\right)=\frac{W_{s}-W_{d}}{W_{d}}\times 100$$

### 2.12. In Vitro Biological Characterization

#### 2.12.1. Rat Cardiac Progenitor Cells Isolation and Culture

Cardiac progenitor cells (rCPCs) were isolated from rats constitutively expressing green fluorescence protein (GFP) [23] and cultured as previously described [24,25]. rCPCs at passages 4 and 5 (P4–P5) were employed for the experiments described in this study.

#### 2.12.2. DiI Cell Labeling

In order to optimize the detection of GFP^pos^ rCPCs cultured on the sponges, cells were stained with CellTracker CM-DiI (ThermoFischer, C-7001) before seeding in agreement with manufacturer instructions [26]. CellTracker CM-DiI is a DiI derivative that is somewhat more water-soluble than DiI, thus facilitating the preparation of staining solutions for cell suspensions. CellTracker CM-DiI contains a thiol-reactive chloromethyl moiety (CM) that allows the dye to covalently bind to cellular thiols. Thus, unlike other membrane stains, this label is well retained in the cells throughout several mitotic divisions, and cell-to-cell contact does not allow dye diffusion.

#### 2.12.3. Cell Culture on AGE Scaffolds

AGE scaffolds were sterilized by UV exposure for 15 min on each side, and cells were seeded on their surface at 45 × 10^3^ cells/cm^2^ density, according to a previously described methodology [21].

Cells loaded to CTRL, MIP-GRGDSP, and MIP-YIGSR sponges were evaluated 72 h after cell plating. The quantification of cell adhesion was performed by “Image Pro Plus 4.0” software [26].

### 2.13. In Vivo Biological Characterization

In order to test the in vivo properties of functionalized AGE scaffolds, experiments were performed on a cryoinjury (CI) rat model. In particular, the suturability, cell adhesion to the sponges, cell migration toward the scaffolds, and cell differentiation were evaluated.

The study population consisted of male Wistar rats (Rattus norvegicus, Charles River, Italy) bred at the University of Parma departmental animal facility. The investigation was conformed to the National Ethical Guidelines (Italian Ministry of Health; D.L.vo 116, January 27, 1992) and the Guide for the Care and Use of Laboratory Animals (NIH publication no. 85–23, revised 1996) and approved by the Veterinary Animal Care and Use Committee of the University of Parma.

In vivo studies in the CI model were performed employing AGE not functionalized (NF) sponges or functionalized with MIP-GRGDSP and MIP-YIGSR sequences. rCPC-seeded (+rCPCs) or unseeded configurations were tested. Each experimental group consisted of four animals.

Cell seeding was performed as described in the previous paragraph. After 48 h, AGE + rCPCs were surgically sutured to cover the damaged area of the rat hearts.

The control group was represented by animals subjected to myocardial damage without scaffolds application.

#### 2.13.1. Surgical Procedure

The surgical procedure and the subsequent macroscopic examination of the rat myocardium were performed according to a methodology well established in our laboratory and detailed in several publications [21,27,28,29].

At sacrifice, the heart was excised and fixed in 10% formalin. After 24 h, LV transverse sections corresponding to the base, equatorial portion, and apex were embedded in paraffin, and 5-μm-thick sections were cut for immunohistochemical studies.

#### 2.13.2. Immunohistochemical Analysis

The adhesion of GFP^pos^ rCPCs seeded on scaffolds and their differentiation toward cardiomyogenic phenotypes were determined by the immunofluorescence detection of GFP, alpha-sarcomeric actin (α-SARC), which is typically expressed by cardiomyocytes, and von Willebrand Factor (vWF), which is a specific endothelial cell marker. Moreover, unseeded sponge colonization by resident myocardial cell was evaluated by β1-integrin expression.

For these purposes, LV sections from different experimental groups were incubated with primary antibodies (polyclonal goat anti-GFP (ab6673), dilution 1:100, Abcam; monoclonal mouse anti-α-SARC (A2172), dilution 1:100, Sigma Aldrich; polyclonal rabbit anti-vWF (A0082), dilution 1:100, Dako; monoclonal rabbit anti- β1-integrin (ab52971), dilution 1:250, Abcam). FITC- and TRITC-conjugated specific secondary antibodies were used to simultaneously detect epitopes. 

Nuclei were recognized by the blue fluorescence of 4′,6-diamindine-2-phenyndole (DAPI, Sigma Aldrich, Milan, Italy) staining.

#### 2.13.3. Data Management and Statistics

The SPSS statistical software was employed (SPSS, Chicago, IL, USA) to perform data management and statistics. A paired Student *t*-test and one-way analysis of variance (ANOVA, post-hoc analyses: Tukey test or Holm–Sidak test) were applied. Statistical significance was set at *p*  <  0.05.

## 3. Results and Discussion

### 3.1. MIP Characterization

#### 3.1.1. Morphological Analysis by Scanning Electron Microscopy

**Figure 1:**
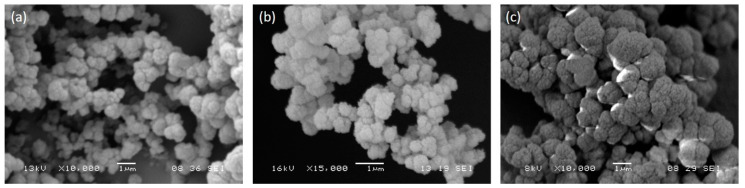
SEM micrographs of MIP-GRGDSP (**a**), MIP-YIGSR (**b**), and CP (**c**). Images were acquired at different magnifications, as reported by scale bars. CP: control particles, MIP: molecularly imprinted particles.

The morphology of MIP and CP was examined by SEM. Micrographs (Figure 1) showed the formation of densely fused microgel particles, with a spherical shape and an average diameter (measured by SEM software) of about 1 µm. No significant difference was observed between MIP and CP in terms of shape and size, but we observed that the presence of the peptide increased particle aggregation. The nature of the template (GRGDSP or YIGSR) did not modify the morphology of imprinted particles.

In consideration of the aggregated conformation, particles were crushed in a mortar before use.

#### 3.1.2. Chromatographic Analysis

Monomer conversion, the amount of template entrapped by the polymer, and the amount of extracted template were determined by HPLC. Results are collected in Table 2. A very high monomer conversion was observed during the polymerization of both MIP-GRGDSP and MIP-YIGSR, indicating that the reaction went to completion.

The amount of template contained in the final product, which was calculated as the difference between the amounts contained in the polymerization solution before and after polymerization, was very high for all the imprinted particles.

The removal of template from the particles was not complete, probably as a consequence of the high cross-linking degree and of the rigidity of the matrix. As reported in the literature [30], bulky templates cannot be easily extracted from polymer networks. Additionally, interactions between the monomer and the template are established, and they can further reduce the efficacy of the extraction procedure. The GRGDSP peptide has two acid groups and two basic groups, and therefore, the molecule is neutral; the interactions with the monomer depend on the delocalization of the negative charge that occurs in the monomer when it is introduced in the ACN/water solution. In this condition, ionic bonds are established between the monomer and the basic groups of the GRGDSP peptide. In the case of the YIGSR peptide, the presence of Tyr residue in the peptide molecule permitted the creation of ionic bonds with the monomer.

#### 3.1.3. Infrared Spectroscopy

**Figure 2:**
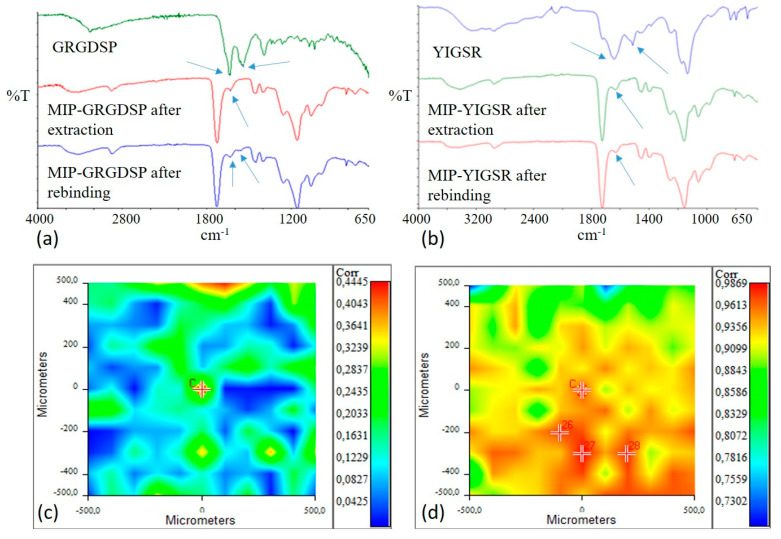
Infrared spectra of: (**a**) GRGDSP peptide, MIP-GRGDSP after extraction, and MIP-GRGDSP after rebinding; (**b**) YIGSR, MIP-YIGSR after extraction, and MIP-YIGSR after rebinding. Amide I and Amide II adsorption bands are indicated by arrows. Correlation maps with YIGSR spectrum for MIP-YIGSR after extraction (**c**) and MIP-YIGSR after rebinding (**d**). Colors in correlation maps correspond to correlation values (from red, higher values, to blue, lower values), as reported in the bars on the right in figures (**c**,**d**).

Infrared spectra of the peptide sequences used as template of MIP after extraction and MIP after the rebinding process were acquired and collected in Figure 2.

GRGDSP and YIGSR spectra were characterized by the presence of bands due to Amide I (1630–1650 cm^−1^) and to Amide II (1550 cm^−1^). The typical bands of MIP polymeric matrix were evident at 3000–2800 cm^−1^ (CH_x_), at 1800–1700 cm^−1^ (C=O), and at 1200–1100 cm^−1^ ((C=O)-O).

In the infrared spectrum of extracted MIP, the band due to Amide I was still present, even if with a lower intensity, confirming that template extraction was not complete, as already observed through the chromatographic analysis.

After the rebinding process, both adsorption bands of the peptide were present in MIP-GRGDSP spectrum, confirming the successful rebinding of the template molecule. In the spectrum acquired for MIP-YIGSR after the rebinding process, only an Amide I adsorption band was evident, while an Amide II band was not appreciable.

Therefore, in order to further verify the absence or the presence of the template in extracted and rebound MIP-YIGSR, a Chemical Imaging investigation was performed in µATR mode.

Correlation maps with YIGSR spectrum (in the range of Amide I and Amide II adsorptions) were acquired for both extracted and rebound MIP-YIGSR particles and collected in Figure 2c-d. For the extracted sample, the value of correlation was low, confirming a good even if not complete extraction of the template from the surface of particle aggregates. Oppositely, the correlation was close to 1 after the rebinding process, confirming the success of the recognition and rebinding process. Moreover, the high correlation value could be indicative of a homogeneous distribution of the rebinding cavities on the MIP surface.

#### 3.1.4. Recognition and Selectivity Tests

Results of the recognition and selectivity tests are collected in Table 3. The molecules used as analogues were GRGDSP for MIP-YIGSR and YIGSR for MIP-GRGDSP.

Values of quantitative binding, which is the amount of template bound per mass of polymer, were higher than those generally obtained with both peptide templates [4,5] and more rigid and smaller templates [31]. The values of unspecific adsorption (which is the amount of template bound by CP) were also high. This can be considered a consequence of the interactions that can be created between the particle matrix and the template molecules, as described under the chromatographic analysis section. Unspecific binding is one of the challenges commonly reported in the literature for protein-imprinted materials [32]. However, in this paper, the ability of MIP to recognize and rebind the template better than control was demonstrated by the values of the recognition factor (ratio between template bound by MIP and template bound by CP), which were higher than one for both MIP systems.

With specific reference to MIP-YIGSR, comparing the results obtained in this work with those previously reported [5], the quantitative binding was increased, while the percentage of unspecific adsorption with respect to total adsorption (template bound by CP/template bound by MIP) was significantly decreased (from 83.3% in [5] to 60.0% in this work). As a consequence, an increase in the recognition factor (1.66 against 1.2 in [5]) was reported. These results could be explained by the different cross-linker used. As already reported in the literature [33], template affinity is greatly influenced by the nature of the cross-linker, and in particular, recognition properties are improved when PETRA is used instead of trimethylpropane trimethacrylate.

The ability of the prepared MIP to selectively recognize the template molecule or a similar peptide was investigated through the determination of the selectivity factor, which is the ratio between the template bound by MIP and analogue bound by MIP, using the same rebinding procedure. As shown in Table 3, both MIP systems were able to distinguish between the template and an analogue molecule, with a selectivity factor much higher than one.

### 3.2. AGE Sponges Characterization

As the AGE sponges are new scaffolds, developed for the first time in this work, their physicochemical and mechanical properties were first investigated before performing functionalization. The results reported in the next sections are not influenced by the subsequent deposition of MIP particles, as preliminarily verified by us.

#### 3.2.1. Morphological Analysis

**Figure 3:**
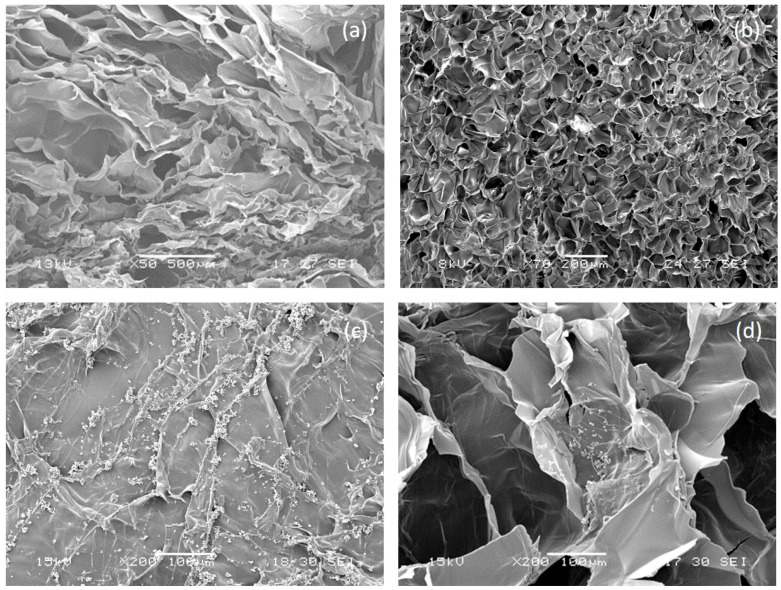
Morphological analysis of alginate/gelatin/elastin (AGE) sponges. Section and surface of not-functionalized AGE sponge are shown in (**a**,**b**), respectively; section and surface of MIP-functionalized AGE sponge are shown in (**c**,**d**), respectively.

SEM micrographs of not functionalized AGE sponge (Figure 3a,b) showed a homogeneous and highly porous structure, with well interconnected pores. The average pore size and porosity percentage, as calculated by Image J analysis of SEM images, were respectively 108 ± 10 µm and 62.5 ± 0.1%. Previous studies showed that spongy scaffolds based on natural polymers with similar porosity properties are able to promote cardiac tissue growth [34].

#### 3.2.2. Infrared Analysis

Infrared spectra were acquired for AGE sponges and for pure components (alginate, gelatin, and elastin) and compared. Results are collected in Table 4. The infrared spectra pointed out the typical absorption peaks of the three components: for elastin, 1640 cm^−1^ (Amide I) and 1390 cm^−1^ (Amide II); for gelatin, 1630 cm^−1^ (Amide I); for alginate, 1024 cm^−1^ (C-O-C).

**Figure 4:**
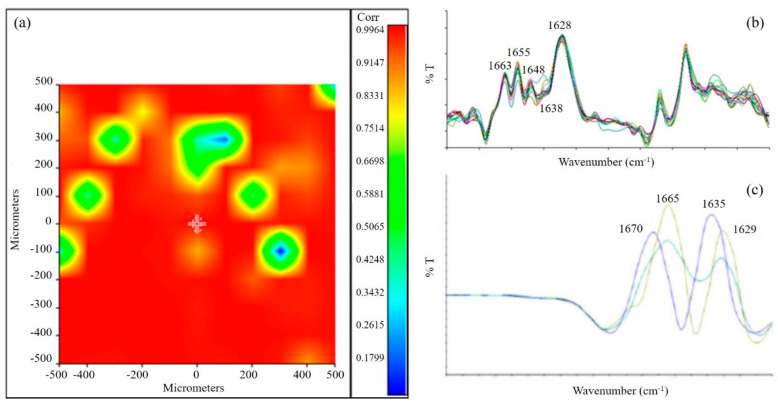
(**a**) Correlation map between the chemical map and the medium spectrum. The green areas in the correlation map are the pores of the sponge; (**b**) Second derivative of AGE spectrum; (**c**) Second derivative of gelatin spectrum.

The chemical map of the sponge was acquired in µ-ATR. The medium spectrum of the chemical map showed the presence of all the typical absorption peaks of the three biopolymers. In addition, the displacement of the typical absorption peak of elastin toward a higher frequency (from 1390 to 1400 cm^−1^), as well as the displacement of the typical band of alginate (from 1024 to 1033 cm^−1^), suggested the presence of interactions (hydrogen bond type) between the protein and the polysaccharide components [35], which were already observed for a scaffold based on blends of protein and polysaccharide polymers [18]. The correlation map between the chemical map and the medium spectrum showed a value around 1 for all the analyzed samples, demonstrating a good chemical homogeneity (Figure 4a). The advantage offered by the homogeneous distribution of scaffold components is to avoid the presence of regions with different characteristics, so as to have a homogenous effect on cell response. Moreover, the homogeneous distribution of the different components within the scaffold mimics the characteristic properties of native cardiac tissue, as reported in the literature [18].

Second derivative analysis was also performed on the scaffold to investigate the secondary structure of the protein component [36]. Spectra collected from the second derivative map of the AGE scaffold showed the following peaks (Figure 4b): at 1663 cm^−1^, due to β-turn conformation; at 1655 cm^−1^ and 1648 cm^−1^, due to α-helix; at 1638 cm^−1^, due to triple helix; at 1628 cm^−1^, due to a β-sheet or random coil structure. These peaks are similar to those observed in the literature for the native tissue [37]. Oppositely, second derivative analysis performed on pure gelatin (Figure 4c) showed a high band variability. These results suggested that the interactions established as a consequence of gelatin blending with alginate and elastin resulted in a partial reorganization of the protein material.

#### 3.2.3. Thermal Analysis

**Figure 5:**
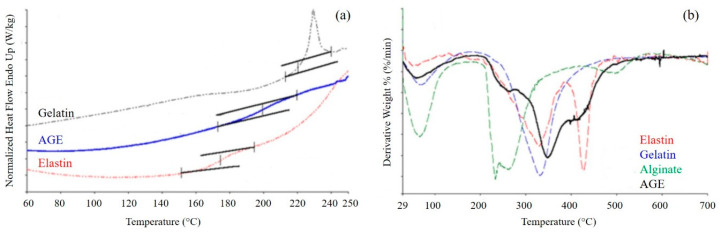
(**a**) Differential scanning calorimetry (DSC) thermograms of gelatin, elastin, and AGE blend. Inflections in the curves indicate a glass transition event; (**b**) Derivative curves obtained by thermogravimetric analysis of AGE blend and pure components. Peaks in the curves indicate events of weight loss.

Thermal properties of the AGE scaffold were investigated by DSC and TGA (Figure 5). Variations in the thermal properties and in the thermodegradation behavior of the blend with respect to pure polymers can be considered an experimental evidence of the presence of interactions among the typical functional groups of pure polymers [35].

DSC thermograms (Figure 5a) were acquired for the sponge, as well as for the single components. Gelatin presented a T_g_ between 220 and 250 °C, and elastin showed a T_g_ between 160 and 200 °C. Alginate thermogram is not reported, since it was characterized only by the thermal degradation of the polymer, which occurred at about 300 °C. The blend displayed only one glass transition event, at a temperature intermediate between those of gelatin and elastin, confirming the presence of strong interactions between the two proteins.

TGA was performed on both the AGE blend and the pure components (Figure 5b). The TGA curve of gelatin showed two events of weight loss: the first one between 30 and 150 °C, with a maximum at 50 °C, due to the loss of water; and the second one between 200 and 500 °C, with a maximum at 329 °C, due to polymer degradation. Alginate showed as well the loss of water, with a maximum at 60 °C, and polymer degradation between 200 and 350 °C. Elastin thermogram displayed three events of weight loss: the loss of water, between 30 and 150 °C, with a maximum at 70 °C; protein degradation, between 150 and 380 °C and a maximum at 330 °C; degradation of the not perfectly hydrolyzed protein, between 380 and 500 °C, with a maximum at 425 °C. The sponge showed two events of weight loss: the first, between 30 and 150 °C, due to the loss of water; the second, between 200 and 500 °C, due to the thermal degradation of the blend and made by the superimposition of the different thermal degradation events of blend components. The presence of only one thermal degradation event further confirmed the presence of strong interactions among the components.

#### 3.2.4. Hydrolytic Degradation Test

Degradation tests on the AGE sponge were performed in PBS, maintaining the samples in an agitating bath at 37 °C. Weight loss was evaluated at appointed times.

**Figure 6:**
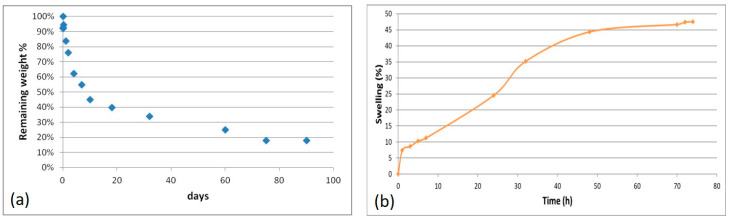
Results of the hydrolytic degradation test (**a**) and swelling test (**b**) performed on AGE sponges.

The results of the hydrolytic degradation test are collected in Figure 6a. Weight loss was quite rapid during the first 7 days (45%), which was probably a consequence of the release of not cross-linked material; then, the process proceeded more slowly, with a remaining weight of 18% after 90 days.

The three natural polymers used for scaffold fabrication are soluble in water. If not cross-linked, the scaffolds would dissolve immediately, after immersion in aqueous environment, and this would hinder cell cultivation and growth on scaffolds. The results obtain in the hydrolytic degradation test showed that the ionic and chemical cross-linking procedures performed on the AGE samples provided them with stability in aqueous environment, without compromising the biodegradability of scaffold components.

#### 3.2.5. Swelling Test

A swelling test was performed by the exposure of AGE sponges to water vapors to evaluate the ability to absorb water. This property is in relation to the material hydrophilicity, and it depends on the chemical properties of blend components, the cross-linking degree, the interactions among components, and the morphology of the scaffold.

The swelling kinetic is shown in Figure 6b. Comparing the obtained results with those already reported in the literature for alginate/gelatin sponges [18], a lower swelling degree and therefore a lower hydrophilicity was observed for AGE sponges. This could be explained considering that elastin is less hydrophilic than gelatin; moreover, the hydrogen bond interactions established between elastin and gelatin involve chemical groups that otherwise could be available to bind water.

#### 3.2.6. Mechanical Analysis

Viscoelastic properties of AGE sponges were investigated by DMA. The results are collected in Table 5.

Both E’ and E’’ values increased with the increase of oscillation frequency, showing an increase of sample stiffness with frequency increase. Values of E’ were higher than values of E’’, demonstrating a predominant elastic behavior.

Comparing these results with those reported in a previous paper for decellularyzed myocardial tissue and sponges based on an alginate/gelatin blend [18], we observed that both values of E’ and E’’ were higher for AGE sponges than for native myocardium. However, a significant decrease of both E’ and E’’ with respect to alginate/gelatin sponges was produced by the addition of the elastin component. This result was expected, since elastin is less stiff than alginate and gelatin [38]. Therefore, even if AGE scaffolds are still stiffer than the native myocardium, their viscoelastic properties are closer to that of the native tissue with respect to sponges based on two components only (alginate and gelatin).

### 3.3. Characterization of MIP Functionalized AGE Sponges

#### 3.3.1. Morphological Analysis

Morphological analysis was repeated after the deposition of imprinted particles (Figure 3c,d). The morphological analysis showed a quite homogeneous distribution of MIP aggregates on the scaffold surface, while a few particles were also present on pore walls. No significant difference was observed in terms of surface distribution between the two different types of particles.

#### 3.3.2. Recognition Experiments

Recognition tests were performed on AGE sponges functionalized by the deposition of MIP-GRGDSP or MIP-YIGSR. Sponges containing CP were used as control. Results are collected in Table 6.

The results showed that the deposition of MIP-GRGDSP and MIP-YIGSR on polymeric sponges did not alter the specific recognition and binding behavior of the imprinted particles.

Moreover, comparing these results with those obtained for free MIP (quantitative binding of 57.34 µmol/g of polymer for MIP-GRGDSP and 83.32 µmol/g of polymer for MIP-YIGSR, see Table 3), sponges functionalized with MIP showed a higher quantitative binding than free particles, suggesting the creation on the sponges of a preferred microenvironment for the rebinding process.

The higher quantitative binding found for scaffolds functionalized with MIP-YIGSR with respect to those functionalized with MIP-GRGDSP could be explained by two factors: (i) the higher percentage of template extraction obtained for MIP-YIGSR than for MIP-GRGDSP, which increases the number of imprinted cavities available to rebind the template; and (ii) the higher unspecific adsorption, demonstrated by the higher quantitative binding on sponges functionalized with CP.

#### 3.3.3. In Vitro and In Vivo Studies

The in vitro quantitative evaluation of the fractional area occupied by DiI-labeled cells documented the presence after 72 h of rCPCs on AGE sponges in both functionalized and not functionalized forms (supplementary Figure S1).

The persistence of rCPCs cultured on AGE scaffolds prompted us to employ the compounds for in vivo testing.

**Figure 7:**
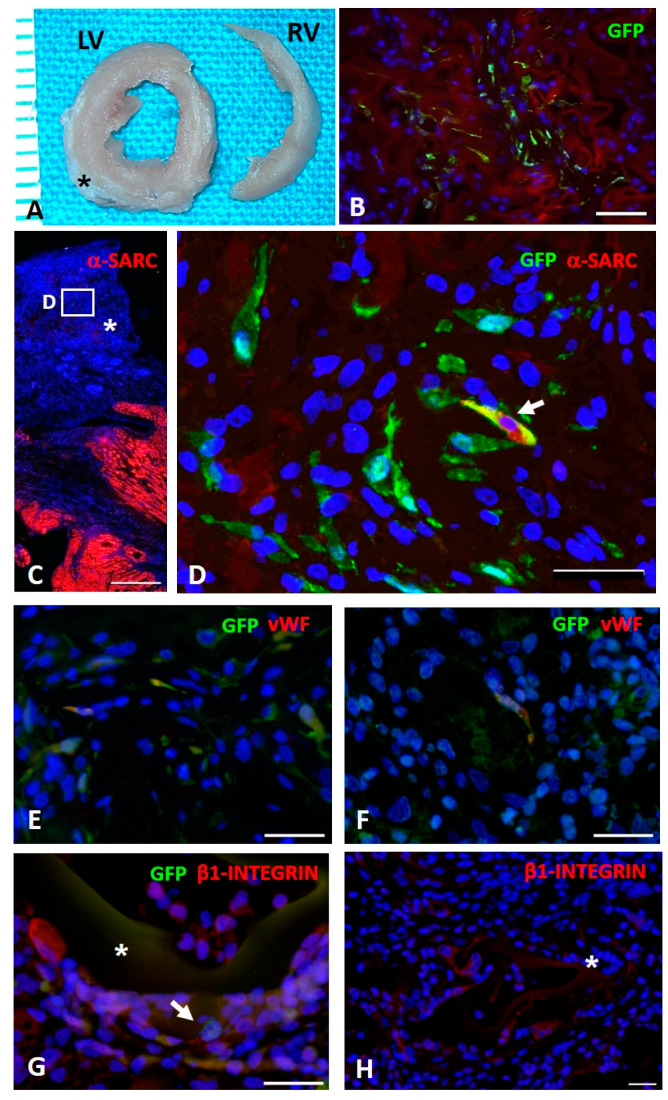
In vivo study. (**A**): Transverse sections of formalin fixed left (LV) and right (RV) ventricles of a cryoinjured rat heart (ten days) in which the sutured AGE sponge (black asterisk) is visible. (**B**): Immunohistochemical detection of green fluorescence protein (GFP^pos^) CPCs (green) seeded on AGE + MIP-GRGDSP sponge sutured on a cryoinjured rat heart. C–F: Immunofluorescence detection of GFP^pos^ CPCs differentiation toward myocardial phenotypes. (**C**): Image taken at low magnification documenting by red fluorescence the spared myocardium labeled by alpha-sarcomeric actin (α-SARC). The asterisk indicates the sutured AGE + MIP-YIGSR sponge in which the white rectangle inscribes the area shown at higher magnification in D. (**D**): GFP^pos^ CPCs (green) located within AGE + MIP-YIGSR sponge sutured to the cryoinjured myocardium. Arrow points to a cell displaying cytoplasmic yellow fluorescence as a result of the co-expression of GFP (green) and α-SARC (red) and documenting the occurrence, after ten days from in vivo implant, of progenitor cells differentiation toward myocardial phenotypes. Green fluorescence labeling depicts several undifferentiated GFP^pos^ α-SARC^neg^ cells. (**E**,**F**): Immunohistochemical analysis of GFP^pos^ rCPCs (green) seeded on AGE + MIP-GRGDSP (**E**) and AGE + MIP-YIGSR (**F**) illustrating by yellowish fluorescence the co-expression of the endothelial marker von Willebrand factor (vWF, red). (**G**): Microscopic image illustrating by yellowish fluorescence the expression of β1-Integrin (red) on GFP^pos^ rCPCs (green, arrow) seeded on AGE + MIP-YIGSR (asterisk) implanted on the cryoinjured myocardium. (**H**): Microphotograph documenting the expression of β1-Integrin in resident myocardial cells located at the edge of unseeded AGE (CTRL) sponge (asterisk). To highlight the presence of β1-Integrin^pos^ fluorescent CPCs, contrast enhancement of the red (TRITC) channel was applied to show the reddish autofluorescence background of the sponge. In B–H, blue fluorescence corresponds to DAPI staining of nuclei. Scale bars: B = 50 µm, C = 500 µm, D, E, F and H = 30 µm; G = 20 µm.

Seeded and unseeded sponges were sutured on the damaged area of rat hearts in all cryoinjured experimental groups (Figure 7A). The immunofluorescence detection of GFP^pos^ rCPCs in the myocardium revealed that progenitor cells were still present within AGE sponges 10 days after their implant (Figure 7B).

Moreover, GFP^pos^ rCPC differentiation toward myocardial adult phenotypes was immunohistochemically detectable. In particular, we documented that MIP-GRGDSP and MIP-YIGSR functionalization fostered the expression of α-SARC and vWF proteins (Figure 7C–F), suggesting an important role of implanted AGE sponges in the differentiation of seeded rCPCs toward myocytes and endothelial cells respectively.

The detection of β1-integrin on GFP^pos^ cells located within the scaffolds supported the hypothesis of the ability of the sponge to actively interact with the native myocardium and promote cell adhesion (Figure 7G). This observation was further confirmed by the observation of β1-integrin^pos^ resident myocardial cells located at the edge of unseeded AGE sponges (Figure 7H).

## 4. Conclusions

The development of scaffolds containing biomolecular signals typically present in the ECM of native tissues is of fundamental importance for successful tissue engineering strategies. Scaffold functionalization with peptides is normally achieved by surface or bulk modification, creating a covalent binding between peptide and polymer functional groups. In the present investigation, we propose an innovative approach for scaffolds functionalization, based on Molecular Imprinting technology, which was reported for the first time by our research group a few years ago. Molecularly imprinted particles with recognition properties toward bioactive molecules of interest (such as peptide sequences) can be deposited on the scaffold surface, where they maintain their recognition properties, promoting an enrichment on the scaffold of a chosen protein and consequently a desired interaction at the cellular and tissue level. In this work, molecularly imprinted particles with recognition properties toward peptide sequences from laminin and fibronectin were obtained by the copolymerization of MAA with the template molecule, in the presence of a cross-linker (PETRA). GRGDSP, from fibronectin, and YIGSR, from laminin, were used as templates. Particles with a spherical shape and an average diameter of 1 µm were obtained for both formulations. High values of monomer conversion and template entrapment were observed. The template molecule was only partially removed from the particles (percentage of extracted template below 50%), as typically occurs with a protein template. Infrared analysis confirmed the desired chemical structure of MIP, as well as successful template rebinding. Good recognition and rebinding capabilities were demonstrated as recognition factor and selectivity factors resulted much higher than one. Therefore, MIP-YIGSR and MIP-GRGDSP were used for the functionalization of an innovative biomimetic scaffold, based on a blend of three natural polymers. This scaffold, obtained by blending a polysaccharide (alginate) and two proteins (gelatin and elastin), represents a valid substitute of native ECM, mimicking its chemical composition and the interactions that occur among components. The results of morphological, physicochemical, functional, and mechanical characterization showed adequate properties of the produced scaffold for bioengineered applications to cardiac repair. Moreover, the results obtained in the characterization of MIP-modified scaffolds show interesting properties of the synthesized MIP as functionalization devices for the development of bioactive scaffolds. Overall, thanks to the combination of a biomimetic polysaccharide/protein substrate with imprinted particles capable of imparting to the system recognition properties, it is possible to get a functionalized scaffold that is able to promote desired cellular responses, which represent an innovative and promising approach in the field of cardiac tissue engineering.

## Figures and Tables

**Table 1:** Synthesis of molecularly imprinted particles. MIP: molecularly imprinted particles; CP: control particles. The template was GRGDSP for MIP-GRGDSP and YIGSR for MIP-YIGSR.

	Template (mg)	MAA (mL)	PETRA (mL)	AIBN (mg)	ACN/ddH_2_O 70/30 (mL)
MIP-GRDGSP	39.39	0.17	2.27	5.1	30
MIP-YIGSR	39.87	0.17	2.27	5.1	30
CP	-	0.17	2.27	5.1	30

**Table 2:** Results of chromatographic analysis carried out on polymerization solution and extraction solution: conversion of monomer, percentage of template molecule entrapped by the polymer, and amount of template molecule extracted by repetitive washings.

	Monomer Conversion (%)	Entrapped Template (%)	Extracted Template
MIP-GRGDSP	98.8	85	30
MIP-YIGSR	99.6	86	40
CP	99.4	-	-

**Table 3:** Results of recognition and selectivity tests, performed on MIP-YIGSR, MIP-GRGDSP, and relative controls (CP-YIGSR and CP-GRGDSP, respectively).

	Template		Analogue	
	Quantitative Binding (μmol/g polymer)	Recognition Factor	Quantitative Binding (μmol/g polymer)	Selectivity Factor
MIP-YIGSR	83.32	1.66	22.13	3.76
CP-YIGSR	50.02			
MIP-GRGDSP	57.34	1.78	14.24	4.03
CP-GRGDSP	37.50			

**Table 4:** Fourier transformed infrared spectroscopy (FT-IR) analysis. Wavelength displacements in AGE scaffolds for the typical absorption bands of alginate, gelatin, and elastin.

	Am I (cm^−1^)	Am II (cm^−1^)	C-O-C (cm^−1^)
Alginate			1024
Gelatin	1630		
Elastin	1640	1390	
AGE	1650–1635	1400	1033

**Table 5:** Storage modulus (E’), loss modulus (E”), and tangent delta (Tan Delta) of AGE sponges, measured by a dynamic mechanical analyzer (DMA) at three different frequencies (1, 3.5, and 10 Hz). The data are reported as medium value ± SD.

	1 Hz	3.5 Hz	10 Hz
E’ (× 10^4^ Pa)	25.1 ± 0.6	26.1 ± 0.2	27.6 ± 0.4
E’’ (× 10^4^ Pa)	1.6 ± 0.1	2.6 ± 0.2	6.6 ± 0.5
Tan Delta (× 10^−3^)	63.8 ± 0.9	100.5 ± 0.9	247.4 ± 0.2

**Table 6:** Results of recognition tests performed on AGE sponges functionalized with MIP-GRGDSP and MIP-YIGSR, and relative controls (sponge + CP).

	Template	
	Quantitative Binding (μmol/g polymer)	Recognition Factor
Sponge + MIP-GRGDSP	72.7	1.99
Sponge + CP	36.6	
Sponge + MIP-YIGSR	203.7	2.09
Sponge + CP	97.3	

## Supplementary Materials

The following are available online at https://www.mdpi.com/2313-7673/5/4/67/s1, Figure S1: Bar Graph showing the in vitro quantitative evaluation of the fractional area occupied by DiI labeled rCPCs on AGE sponges after 72 h of culture.

## References

[1] Tallawi M., Rosellini E., Barbani N., Cascone M.G., Rai R., Saint-Pierre G., Boccaccini A.R. (2015). Strategies for the chemical and biological functionalization of scaffolds for cardiac tissue engineering: A review. J. R. Soc. Interface.

[2] Hynes R.O. (1999). Cell adhesion: Old and new questions. Trends Cell Biol..

[3] Shin H., Jo S., Mikos A.G. (2003). Biomimetic materials for tissue engineering. Biomaterials.

[4] Rosellini E., Barbani N., Giusti P., Ciardelli G., Cristallini C. (2010). Novel Bioactive Scaffolds with Fibronectin Recognition Nanosites Based on Molecular Imprinting Technology. J. Appl. Polym. Sci..

[5] Rosellini E., Barbani N., Giusti P., Ciardelli G., Cristallini C. (2010). Molecularly Imprinted Nanoparticles with Recognition Properties Towards a Laminin H–Tyr–Ile–Gly–Ser–Arg–OH Sequence for Tissue Engineering Applications. Biomed. Mater..

[6] Rachkov A., Minoura N. (2001). Towards molecularly imprinted polymers selective to peptides and proteins. The epitope approach. Biochim. Biophys. Acta.

[7] Akiyama S.K., Yamada K.M. (1987). Fibronectin. Adv. Enzym. Relat. Areas Mol. Biol..

[8] Humphries M.J., Obara M., Olden K., Yamada K.M. (1989). Role of fibronectin in adhesion, migration, and metastasis. Cancer Investig..

[9] Larsen M., Artym V.V., Green J.A., Yamada K.M. (2006). The matrix reorganized: Extracellular matrix remodeling and integrin signaling. Curr. Opin. Cell Biol..

[10] Colognato H., Yurchenco P.D. (2000). Form and function: The laminin family of heterotrimers. Dev. Dyn..

[11] Miner J.H., Yurchenco P.D. (2004). Laminin functions in tissue morphogenesis. Ann. Rev. Cell Dev. Biol..

[12] Hersel U., Dahmen C., Kessler H. (2003). RGD modified polymers: Biomaterials for stimulated cell adhesion and beyond. Biomaterials.

[13] Ross R.S., Borg T.K. (2001). Integrins and the myocardium. Circ. Res..

[14] Maitra N., Flink I.L., Bahl J.J., Morkin E. (2000). Expression of α and β integrins during terminal differentiation of cardiomyocytes. Cardiovasc. Res..

[15] Massia S.P., Hubbell J.A. (1990). Covalent surface immobilization of Arg-Gly-Asp- and Tyr-Ile-Gly-Ser-Arg-containing peptides to obtain well-defined cell-adhesive substrates. Anal. Biochem..

[16] Dee K.C., Andersen T.T., Bizios R. (1994). Cell function on substrates containing immobilized bioactive peptides. Mater. Res. Soc. Symp. Proc..

[17] Battista S., Guarnieri D., Borselli C., Zeppetelli S., Borzacchiello A., Mayol L., Gerbasio D., Keene D.R., Ambrosio L., Netti P.A. (2005). The effect of matrix composition of 3D constructs on embryonic stem cell differentiation. Biomaterials.

[18] Rosellini E., Zhang Y.S., Migliori B., Barbani N., Lazzeri L., Shin S.R., Dokmeci M.R., Cascone M.G. (2018). Protein/Polysaccharide-based Scaffolds Mimicking Native Extracellular Matrix for Cardiac Tissue Engineering Applications. J. Biomed. Mater. Res. A.

[19] Massia S.P., Hubbell J.A. (1991). An RGD spacing of 440 nm is sufficient for integrin αVβ3-mediated fibroblast spreading and 140 nm for focal contact and stress fiber formation. J. Cell Biol..

[20] Rowley J.A., Madlambayan G., Mooney D.J. (1999). Alginate hydrogels as synthetic extracellular matrix materials. Biomaterials.

[21] Frati C., Graiani G., Barbani N., Madeddu D., Falco A., Quaini F., Lazzeri L., Cascone M.G., Rosellini E. (2020). Reinforced alginate/gelatin sponges functionalized by avidin/biotin-binding strategy: A novel cardiac patch. J. Biomater. Appl..

[22] ISO 10993-13 (2010). Biological Evaluation of Medical Devices Identification and Quantification of Degradation Products from Polymeric Medical Devices.

[23] Okabe M., Ikawa M., Kominami K., Nakanishi T., Nishimune Y. (1997). Green mice as a source of ubiquitous green cells. FEBS Lett..

[24] Frati C., Savi M., Graiani G., Lagrasta C., Cavalli S., Prezioso L., Rosseti P., Mangiaracina C., Ferrado F., Madeddu D. (2011). Resident cardiac stem cells. Curr. Pharm. Des..

[25] Giuliani A., Frati C., Rossini A., Komlev V.S., Lagrasta C., Savi M., Cavalli S., Gaetano C., Quaini F., Manescu A. (2011). High-resolution X-ray microtomography for three-dimensional imaging of cardiac progenitor cell homing in infarcted rat hearts. J. Tissue Eng. Regen. Med..

[26] Rai R., Tallawi M., Barbani N., Frati C., Madeddu D., Cavalli S., Graiani G., Quaini F., Roether J.A., Schubert D.W. (2013). Biomimetic poly(glycerol sebacate) (PGS) membranes for cardiac patch application. Mater. Sci. Eng. C Mater. Biol. Appl..

[27] Rossini A., Frati C., Lagrasta C., Graiani G., Scopece A., Cavalli S., Musso E., Baccarin M., Di Segni M., Fagnoni F. (2011). Human cardiac and bone marrow stromal cells exhibit distinctive properties related to their origin. Cardiovasc. Res..

[28] Bocchi L., Savi M., Graiani G., Rossi S., Agnetti A., Stillitano F., Lagrasta C., Baruffi S., Berni R., Frati C. (2011). Growth factor-induced mobilization of cardiac progenitor cells reduces the risk of arrhythmias, in a rat model of chronic myocardial infarction. PLoS ONE.

[29] Savi M., Bocchi L., Rossi S., Frati C., Graiani G., Lagrasta C., Miragoli M., Di Pasquale E., Stirparo G.G., Mastrototaro G. (2016). Antiarrhythmic effect of growth factor-supplemented cardiac progenitor cells in chronic infarcted heart. Am. J. Physiol. Heart Circ. Physiol..

[30] Lorenzo R.A., Carro A.M., Alvarez-Lorenzo C., Concheiro A. (2011). To Remove or Not to Remove? The Challenge of Extracting the Template to Make the Cavities Available in Molecularly Imprinted Polymers (MIPs). Int. J. Mol. Sci..

[31] Ciardelli G., Cioni B., Cristallini C., Barbani N., Silvestri D. (2004). and Giusti, P. Acrylic polymeric nanospheres for the release and recognition of molecules of clinical interest. Biosens. Bioelectron..

[32] Brown M.E., Puleo D.A. (2008). Protein binding to peptide-imprinted porous silica scaffolds. Chem. Eng. J..

[33] Rechichi A., Cristallini C., Vitale U., Ciardelli G., Barbani N., Vozzi G., Giusti P. (2007). New biomedical devices with selective peptide recognition properties. Part 1: Characterization and cytotoxicity of molecularly imprinted polymers. J. Cell. Mol. Med..

[34] Shachar M., Cohen S. (2003). Cardiac Tissue Engineering, Ex-Vivo: Design Principles in Biomaterials and Bioreactors. Heart Fail. Rev..

[35] Daniliuc L., De Kasel C., David C. (1992). Intermolecular interactions in blends of poly(vinyl alcohol) with poly(acrylic acid)-1. FTIR and DSC studies. Eur. Polym. J..

[36] Segtnan V.H., Isaksson T. (2004). Temperature, sample and time dependant structural characteristics of gelatin gels studied by near infrared spectroscopy. Food Hydrocoll..

[37] Liu K.-Z., Jackson M., Sowa M.G., Ju H., Dixon I.M., Mantsch H.H. (1996). Modification of the extracellular matrix following myocardial infarction monitored by FTIR spectroscopy. Biochim. Biophys. Acta.

[38] Grover C.N., Cameron R.E., Best S.M. (2012). Investigating the morphological, mechanical and degradation properties of scaffolds comprising collagen, gelatin and elastin for use in soft tissue engineering. J. Mech. Behav. Biomed..

